# Molecular imaging using the theranostic agent ^197(m)^Hg: phantom measurements and Monte Carlo simulations

**DOI:** 10.1186/s40658-018-0216-9

**Published:** 2018-08-27

**Authors:** Robert Freudenberg, Rudi Apolle, Martin Walther, Holger Hartmann, Jörg Kotzerke

**Affiliations:** 1 Department of Nuclear Medicine, University Hospital Carl Gustav Carus, Fetscherstraße 74, 01307 Dresden, Germany; 2 Institute of Radiooncology – OncoRay, Helmholtz-Zentrum Dresden-Rossendorf, Bautzner Landstraße 400, 01328 Dresden, Germany; 3 Institute of Radiopharmaceutical Cancer Research, Helmholtz-Zentrum Dresden-Rossendorf, Bautzner Landstraße 400, 01328 Dresden, Germany

**Keywords:** GATE, Gamma camera, ^197(m)^Hg, Monte Carlo simulation, Radiomercury, Journal: EJNMMI Physics

## Abstract

**Background:**

Radiomercury ^197m^Hg and ^197^Hg, henceforth referred to as ^197(m)^Hg, is a promising theranostic radionuclide endowed with properties that allow diagnostic and therapeutic applications. The aim of this work was to investigate the capabilities of ^197(m)^Hg for nuclear medicine imaging. Therefore measurements were performed by using a Philips BrightView SPECT camera. Furthermore, Monte Carlo simulations using the GATE software were performed to theoretically explore the imaging contribution from the various gamma and X-ray emissions from ^197(m)^Hg for a commercial clinical camera with low-energy high-resolution (LEHR) and high-energy general-purpose (HEGP) collimators. We estimated the spatial resolution by using a four-quadrant bar phantom, and we evaluated the planar and tomographic images from an abdominal phantom containing three cylindrical sources of ^197(m)^Hg solution.

**Results:**

A good accordance between measurements and simulations was found for planar and SPECT imaging. Simulations allowed the decomposition of the detected energy spectrum into photon origins. Measurements and simulations for the bar phantom revealed that for the LEHR collimator, the 6-mm pattern could be resolved, whereas for the HEGP collimator, the resolution is about 10 mm. Furthermore, we found that no significant image distortion results from high-energy photons when using the LEHR collimator.

**Conclusions:**

We demonstrated the imaging capabilities of ^197(m)^Hg which is essential both for diagnostic applications and to determine the in vivo biodistribution for dose calculations in therapeutic applications.

## Background

The physical fundament of nuclear medicine is the application of radioactive isotopes. The emitted radiation can be used either for diagnostic or for therapeutic purposes. For diagnostics, it is favorable that the isotopes emit gamma radiation with energies of a few hundred kiloelectron volt like ^99m^Tc, ^123^I, or ^111^In. For therapeutic application, alpha- or beta-particle emitting radionuclides are applied to achieve a large dose deposition in tumors or metastases like ^223^Ra, ^90^Y, ^177^Lu, and ^188^Re.

Prior to therapeutic treatment, it is mandatory to estimate the absorbed dose. To this end, pre-therapeutic imaging is performed in order to visualize the distribution of lesions and to estimate the biokinetics of the radionuclides. It is favorable that the radionuclides for diagnostic and therapeutic application are isotopes of the same chemical element because little variations in the chemical composition of radiotracers can cause large variations in the biodistribution during diagnostic and therapeutic application. Hence, a special focus is on the development of “theranostic” radioisotopes that combine the advantages of therapeutic and diagnostic radioisotopes.

One such radioisotope is mercury ^197^Hg and its metastable state ^197m^Hg, henceforth referred to as ^197(m)^Hg. Radiomercury in the form of ^203^Hg was initially investigated for clinical use in the 1950s [1, 2] and a few diagnostic applications of ^197^Hg occurred in the 1970s [3, 4]. Back then, radiomercury was produced using reactor neutrons and had a low specific activity. Consequently, a large amount of mercury was required for imaging, leading to neurotoxic side effects [5, 6]. The novel possibility of producing ^197(m)^Hg using a cyclotron allows to achieve high specific activities without any neuro- or chemotoxic effects [7]. Hence, ^197(m)^Hg is very attractive for therapeutic and diagnostic application.

Therapeutic applications of ^197m^Hg and ^197^Hg are conceivable because of their suitable emission spectra [8]. The radial dose distribution is comparable to the dose distribution from ^177^Lu [7], a therapeutic radionuclide that is routinely used in nuclear medicine [9]. Apart from its therapeutic potential, ^197m^Hg is suitable for imaging studies due to gamma and X-ray emission upon its decay. This could, for example, allow measurements of its biodistribution, a prerequisite for dose calculations in the therapeutic setting.

This manuscript focuses on the imaging capabilities of ^197(m)^Hg. To evaluate the potential for diagnostic imaging, phantom measurements were performed and compared to Monte Carlo simulations in order to better understand the physical behavior of the photons emitted by ^197(m)^Hg. This is the first step for future research and the transfer to in vitro and in vivo application of ^197(m)^Hg-labeled radiotracers.

## Methods

### ^197^Hg and ^197m^Hg

Mercury-197 exists in two energy states [10]: a longer-lived ground state (^197^Hg, half-life T_1/2_ = 64.1 h) and a shorter-lived excited state (^197m^Hg, T_1/2_ = 23.8 h) separated by an energy difference of 299 keV. ^197^Hg predominantly decays to stable ^197^Au via electron capture, while ^197m^Hg decays either to ^197^Hg or ^197^Au with respective branching ratios of 91 and 9%.

**Fig. 1:**
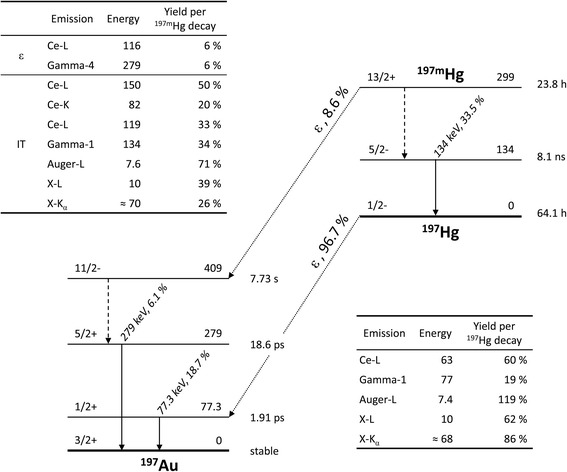
Decay scheme. Level scheme and the most relevant emissions from ^197m^Hg and ^197^Hg

The ^197(m)^Hg emission spectrum comprises a multitude of X-ray, conversion, and auger electron, as well as a few gamma emissions [10]. Dominant photon emissions are observed at energies of 70–80, 134, and 279 keV. The two higher energy contributions are exclusively associated with gamma emissions from ^197m^Hg, while X-ray and gamma emissions from both isomers populate the low-energy domain. Given the difference in half-lives, one can thus expect the higher energy emissions to disappear over time. Figure 1 comprises the decay scheme and the most relevant emissions from ^197m^Hg and ^197^Hg.

Mercury-197 can be produced with a high specific activity (≈ 500 GBq/μmol) via (p,n) reactions on ^197^Au [7], a reaction which creates both isomers in roughly equal proportions. The two samples used for this study were produced with 10 MeV cyclotron protons and measured on the order of 10 MBq in activity of both isomers together.

High-resolution spectroscopy was performed to determine sample purity and to exclude the presence of other nuclides from cyclotron target like ^194^Au, ^196^Au, or ^198^Au. In particular, the presence of radioactive gold isotopes was found to be five orders of magnitude lower in activity than that of ^197(m)^Hg. From gamma spectroscopy, a transferable activity ratio *R*_*a*_ of the two isomers (*R*_*a*_ = *A*(^197^Hg)/*A*(^197m^Hg)) was determined which can be evolved to different measurement times using the half-lives above.

### Camera model

The GATE (version 7.0) simulation toolkit [11] was used to model the BrightView SPECT system (Philips Healthcare, Best, the Netherlands). It utilizes two camera heads, each equipped with a 9.5-mm-thick NaI(Tl) scintillator measuring 40.6 cm axially by 54 cm trans-axially and instrumented with a 59-element PMT array. Camera heads were modeled as a succession of cuboidal layers, representing from the interior outwards: the collimator, an aluminum cover, the NaI crystal, an optical coupling, and a backscatter compartment (BSC), all encased in lead shielding. The BSC (thickness 150 mm, density 1.05 g/cm^3^) simplifies the rather complicated geometry of the PMTs and associated readout electronics into a homogeneous volume whose material composition was taken from Rault et al. [12], who compare such a BSC with a more detailed geometric model with encouraging agreement. It is required for an accurate representation of photons which were scattered into the scintillator from material behind it, which is of particular importance in studies of ^197(m)^Hg since maximally backscattered 279 keV gammas will be energetically indistinguishable from the direct 134 keV gamma signal.

Collimators were implemented as lead cuboids of the appropriate thickness, and collimator holes were introduced by placing hexagonal openings at intervals which result in the correct septal thickness as specified by the vendor [13]. The length, diameter of the circumscribed circle, and septal thickness of the LEHR collimator were 27.0, 1.22, and 0.152 mm, respectively. Corresponding parameters for the HEGP were 58.4, 3.81, and 1.730 mm.

Sources were modeled as volumes containing uniformly distributed mono-energetic point sources. Four energies were considered and simulated separately: 70, 77, 134, and 279 keV. Simulation results were then weighted according to the intensity per disintegration for each of the two isomers and the activity ratio at the time when measurements for comparison were taken. Simulated datasets could thus be matched to measurements taken at various times without the need for further simulation.

### Phantom measurements

**Fig. 2:**
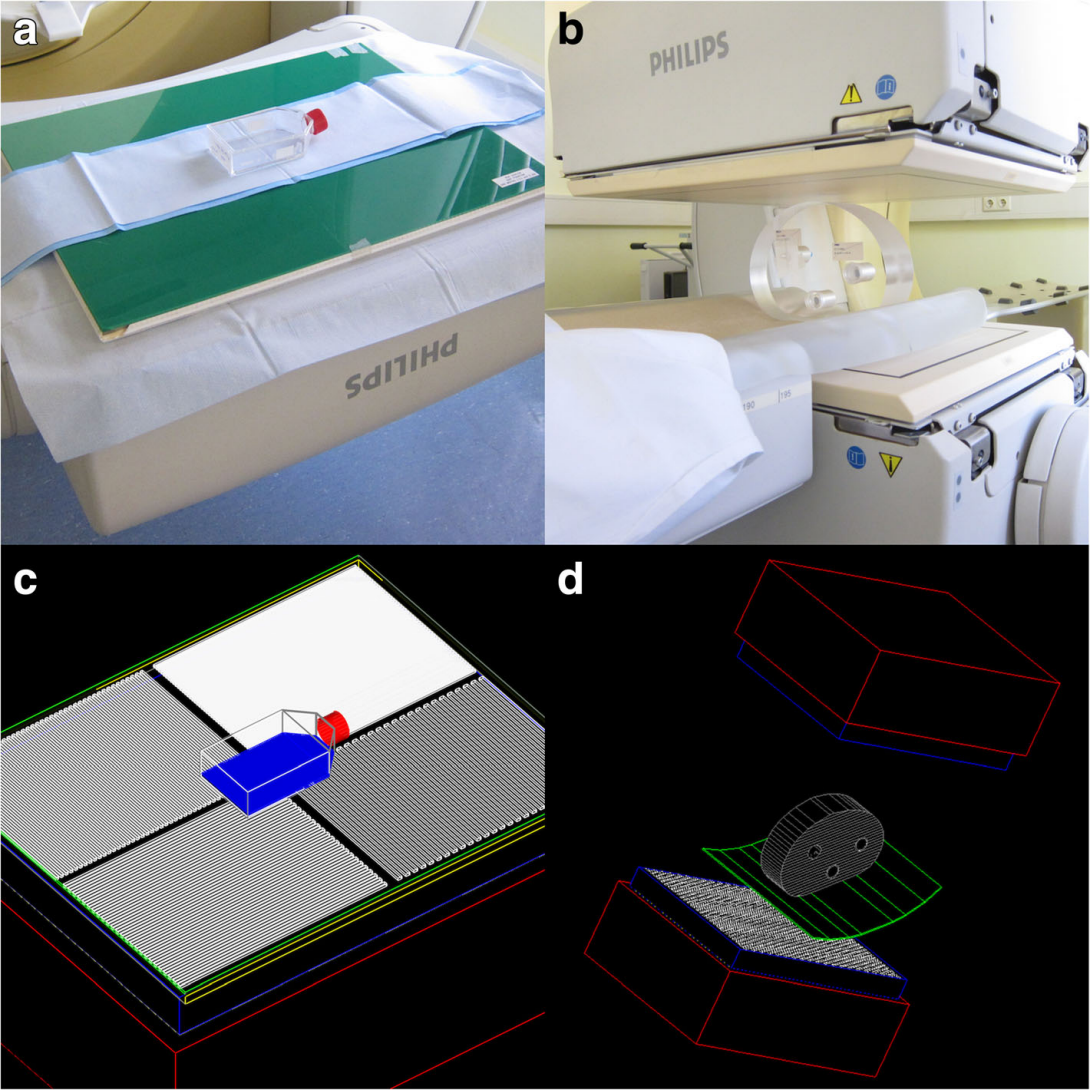
Setup. Measurement setups and corresponding simulated geometries. **a**, **c** Planar setup. **b**, **d** Tomographic setup

Two measurement geometries were investigated: a planar and a tomographic setup. The former utilized a planar source imaged through a Pb-bar phantom (Von Gahlen, Zevenaar, the Netherlands) positioned directly on top of the collimator. The bar phantom comprises four quadrants of different modulation periods: 4, 6, 8, and 10 mm. The source was assembled by filling a 20-ml solution of [^197(m)^Hg]HgCl_2_ in hydrochloric acid into a cell-culture flask measuring approx. 8 cm by 12 cm (Greiner Bio-One, Kremsmünster, Austria), where it formed a 4-mm layer when laying flat. Figure 2a,c illustrates the planar setup and the simulated geometry. Planar acquisitions were then recorded with a square matrix of 1024^2^ pixels measuring 0.314 mm (zoom factor 1.85). Acquisition durations and activity ratios were 151 min and 5.92, respectively, for the LEHR collimator and 165 min and 10.6 for the HEGP model.

The second arrangement uses a polymethyl-methacrylate (PMMA) slab resembling an abdominal section measuring roughly 30 cm by 20 cm by 8 cm (Fig. 2b,d). It contains three hollow cylindrical receptacles, which were loaded with source containers of varying diameter: an 11-ml vial (V11, inner diameter = 19 mm), a 25-ml vial (V25, diameter = 27 mm), and a thin syringe (SYR, diameter = 5 mm). These were filled with various activity concentrations of a sample of activity ratio *R*_*a*_ = 3.7. SPECT acquisitions were then performed with both heads facing one another, equipped with the LEHR collimator and rotating through 180° in 2° intervals held for 5 min each. The acquisition matrix consisted of 128^2^ pixels measuring 3.195 mm (zoom factor 1.46). Tomographic reconstruction was performed using the Astonish algorithm [14] supplied by the vendor which is an extension of OSEM incorporating corrections for collimator resolution at varying head radii. In order to reconstruct simulated and measured data in a uniform fashion, a dummy SPECT dataset was recorded with parameters matching those of the simulation. Simulation results were then embedded into this dataset, uploaded into the vendor-supplied workstation, and reconstructed using the same algorithm. Reconstruction parameters were two subsets and eight iterations in either scenario; scatter and attenuation correction was disabled because ^197(m)^Hg is not listed in the nuclide database.

In order to validate the camera model, especially the BSC, it was necessary to record energy spectra. There was no direct way of obtaining them, but the camera’s acquisition terminal presents a crude spectrum display, a screen shot of which can be analyzed to obtain spectral data. This procedure is limited by the display resolution, which only allowed for a sampling of the spectra with a resolution of 1.05 keV on the energy axis and 45.5 on the count axis. Three separate energy windows were considered for evaluation, with centers and widths of 70 keV (30%), 135 keV (20%), and 280 keV (20%).

### Finite resolution modeling

Two components of system behavior could not be physically simulated by GATE, namely the energy and intrinsic spatial resolution. Both parameters can be considered by digitizer modules in the simulation to model the real behavior event by event. To accelerate the simulations, we considered energy and intrinsic spatial resolution afterwards by convolving the exact energy and position with related blurring functions. The energy blurring determined by measurements of the energy spectra of ^197(m)^Hg and ^131^I samples. The position and width of two dominant photon emissions from each nuclide were fitted using a model comprised of a Gaussian photo-peak on top of a linear background. These results yielded an energy calibration, as well as an energy-dependent resolution function. The energy resolution model was applied to simulation results in two different ways depending on the observable of interest. When examining energy spectra, the simulated spectra were convolved with Gaussian distributions of the appropriate width for the given energy. For investigations of imaging properties, on the other hand, the energies of detected photons and the camera’s ability to resolve them only have an impact on whether an event is registered in a particular energy window or not. The probability of such acceptance was computed as the overlap of the energy window and a cumulative distribution function taking its parameters from the energy resolution model. The event was then weighted accordingly. The intrinsic spatial resolution achieved at relevant ^197(m)^Hg energies was investigated in the planar setup with its modulation patterns of different spatial frequency. Ultimately, an estimate of the line spread function (LSF) was obtained and later used to convolve the simulated images to incorporate the effects of a finite intrinsic spatial resolution. The modulation *M*(*ν*) of a periodic signal at frequency *ν* can be computed as the asymmetry in its maximal and minimal amplitude *M* = (*A*_max_ − *A*_min_)/(*A*_max_ + *A*_min_). Evaluation of the ratio of image (*M*_*i*_) to object modulations (*M*_*o*_) yields the modulation transfer function (*MTF*), which is the LSF’s counterpart in the spatial frequency domain. Its total value can be decomposed into intrinsic and collimator parts as MTF_sys_(*υ*) = MTF_intr_(*υ*) × MTF_coll_(*υ*) [15]. Exploiting this relation and recognizing that the measured MTF includes both components, while the simulation only includes the collimator part, one finds: MTF_intr_(*υ*) = MTF_sys_(*υ*)/MTF_coll_(*υ*) = MTF_data_(*υ*)/MTF_sim_(*υ*), where MTF_data_ and MTF_sim_ are the measured and simulated MTF, respectively. Inserting the definition of modulation transfer (MTF(*υ*) = *M*_*i*_(*υ*)/*M*_*o*_(*υ*)) and assuming that object modulations are equivalent in the simulated and measured cases, one finally obtains

1 $$ {\mathrm{MTF}}_{\mathrm{intr}}\left(\upsilon \right)={M}_{i,\mathrm{data}}\left(\upsilon \right)/{M}_{i,\mathrm{sim}}\left(\upsilon \right) $$

Thus, by determining the image modulation in both measurement and simulation at the four spatial frequencies, the intrinsic MTF can be sampled. Ultimately, a width parameter is sought which can be used in a Gaussian blurring of the simulated images. Assuming the intrinsic spatial resolution to be isotropic, a Gaussian fit to the intrinsic LSF would provide just such a parameter. Hence, a Gaussian approximation of MTF_intr_(*υ*) with width parameter *ω* is found, which can be Fourier transformed into an intrinsic LSF of width *σ*. The two width parameters are related by *σ* = 1/(2*πω*).

**Fig. 3:**
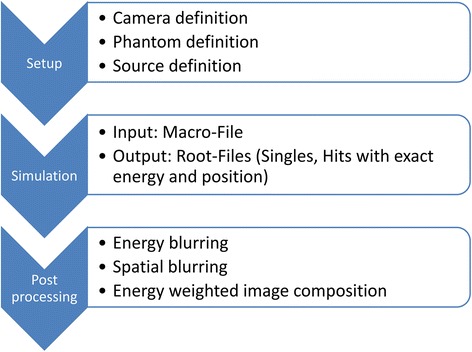
Flow chart. Sequence illustrating the workflow of the GATE simulation and processing

The whole sequence of creating simulation results is illustrated in Fig. 3.

## Results

In each of the following subsections, we will compare the results of measurements and simulations. To clarify our meaning, we stated if we address measurement or simulation.

### Energy spectra

**Fig. 4:**
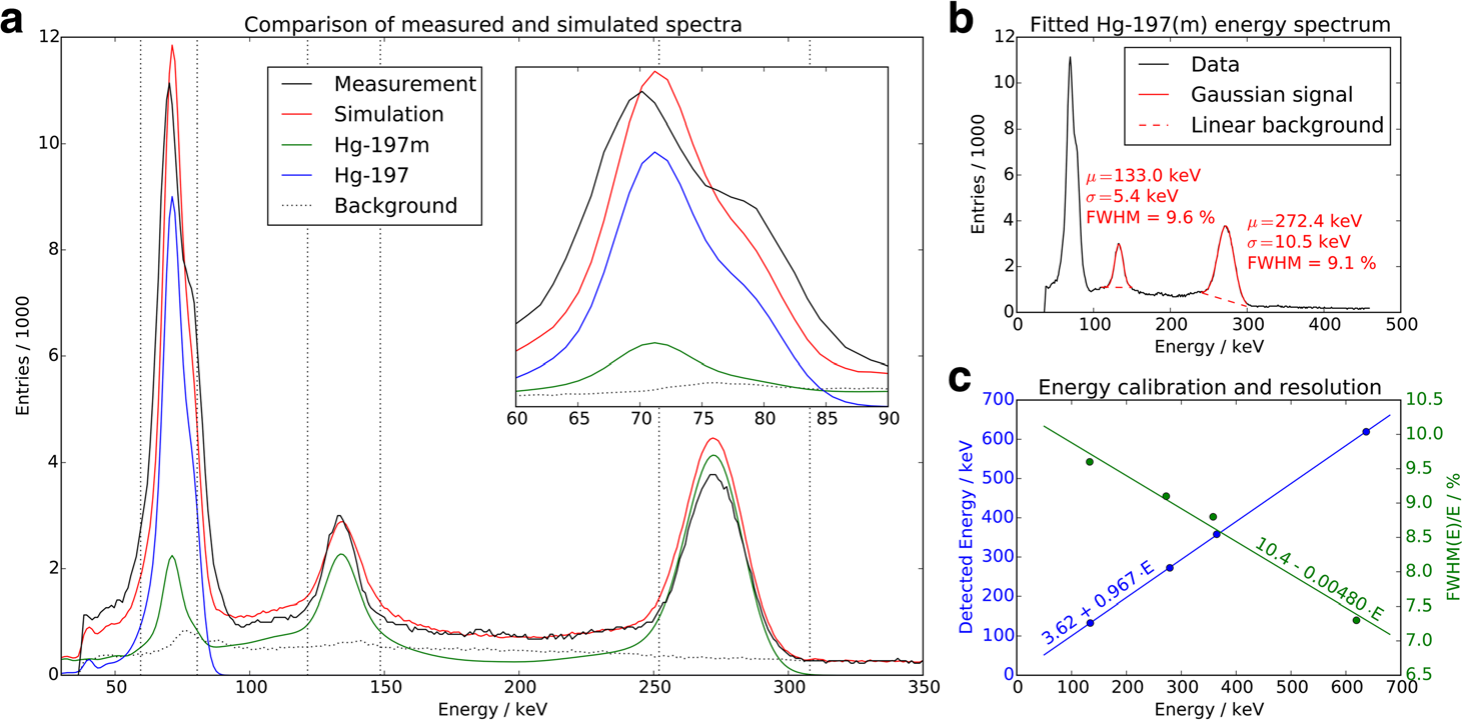
Energy spectra. Investigation of energy spectra. **a** Comparison of measured (black) and simulated (red) energy spectra for a sample with activity ratio *R*_*a*_ = 2.1. The simulated spectrum contains contributions from both isomers and a background template derived from measurement. Energy windows are marked as dotted vertical lines. **b** Measured energy spectrum for ^197(m)^Hg. The center and width of the selected photo-peaks is determined from Gaussian fits and used to derive an energy calibration and determine the energy resolution. **c** Energy calibration (blue) and resolution (green) derived from measurements of each of two ^197(m)^Hg and ^131^I photo-peaks. Linear fits were obtained to interpolate for arbitrary energy values

Figure 4a contrasts the measured and simulated spectra for this sample. The simulated spectrum was supplemented with a measured background distribution whose overall normalization was chosen so as to match the counts in the high-energy sideband (above 320 keV) of the measured spectrum. The simulated contributions of the two isomers were first normalized relative to one another using *R*_*a*_ and then normalized together such that the overall simulated counts match those of the measured spectrum with the normalized background removed.

Relative FWHM energy resolutions measured from the 134- and 279-keV photo-peaks of a ^197(m)^Hg source with *R*_*a*_ = 2.1 were 9.6 and 9.1%, respectively (Fig. 4b). Together with the 365- and 637-keV signals from a measured ^131^I sample, an energy dependence was observed which was linear to a very good approximation (Fig. 4c). The measured resolution compares well to the value stated by the vendor as ≤ 9.6% at 140 keV [13].

**Fig. 5:**
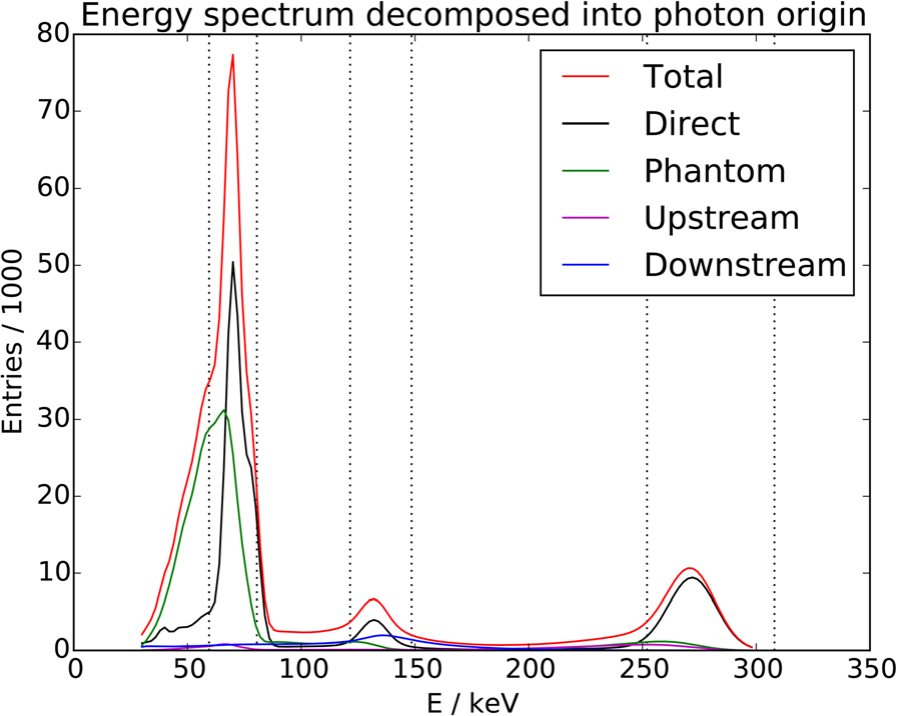
Scatter origins. Energy spectrum simulated in tomographic setup decomposed into direct and inelastically scattered photons. Scatter volumes are classified as phantom (phantom, table), upstream (collimator, crystal cover), or downstream (light-guide, backscatter compartment, shielding). Energy windows are marked as dotted vertical lines

Photon scattering in different compartments was obtained by separately tallying the different events in the simulation. Using this information, the simulated spectrum can be decomposed into photon origins. This is done for a spectrum in the tomographic setup in Fig. 5 in which the energy windows are also indicated. The window at 70 keV contains significant amounts of scattered photons, as does the one at 135 keV. Their sources are different, however. While the former window is mostly scattered into from the phantom or table, the scatter contribution in the latter primarily originates from behind the NaI crystal. The window at 280 keV is largely free of scattered events.

### Planar imaging

**Fig. 6:**
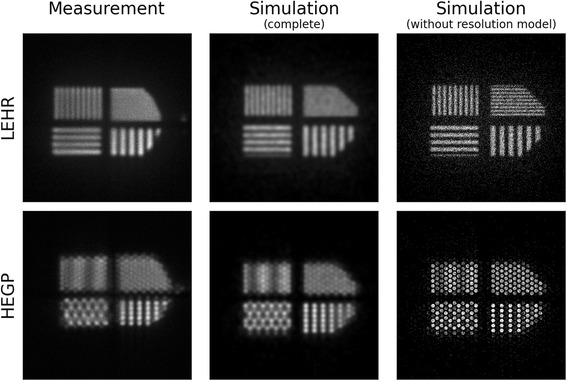
Images of bar phantom. Measured and simulated images in planar setup for two collimators: top: low-energy, high-resolution collimator; bottom: high-energy, general-purpose collimator. Simulated images are shown before (right column) and after (central column) the spatial resolution model has been applied

A comparison of measured and simulated images acquired with the bar phantom and both collimators can be seen in Fig. 6. Both collimators are capable of resolving the lowest spatial frequency segments of the bar pattern, while the highest frequency parts are not resolved. This enables one to find crude resolution limits by inspection. They are located at 6 and 10 mm for the LEHR and HEGP collimators, respectively, at small source-to-collimator distances. The figure also shows the raw simulation results in the rightmost column, which do not yet incorporate the intrinsic resolution model. Even the finest modulation pattern can be clearly resolved with the LEHR collimator, while imaging with the HEGP collimator mostly resolves its hexagonal hole pattern.

The procedure for determining the intrinsic MTF is now employed using measured and simulated LEHR dataset recorded in the energy window centered on 70 keV. First, the individual modulation patterns are projected onto a line running along the direction of modulation yielding profiles through the modulation patterns. Minimal and maximal amplitudes were taken from Gaussian fits to the profiles’ peaks and troughs to smooth out statistical fluctuations. This was repeated for the four modulation patterns and the intrinsic MTF calculated via Eq. 1. The approximated intrinsic MTF was parameterized by a width parameter *ω* = 0.0873 mm^− 1^, which corresponds to an FWHM in the spatial domain of 4.29 mm (*σ* = 1.82 mm). The results of applying this spatial resolution model on the raw simulated data can be seen in the middle column of Fig. 6, which now shows an encouraging correspondence to the measured images.

**Fig. 7:**
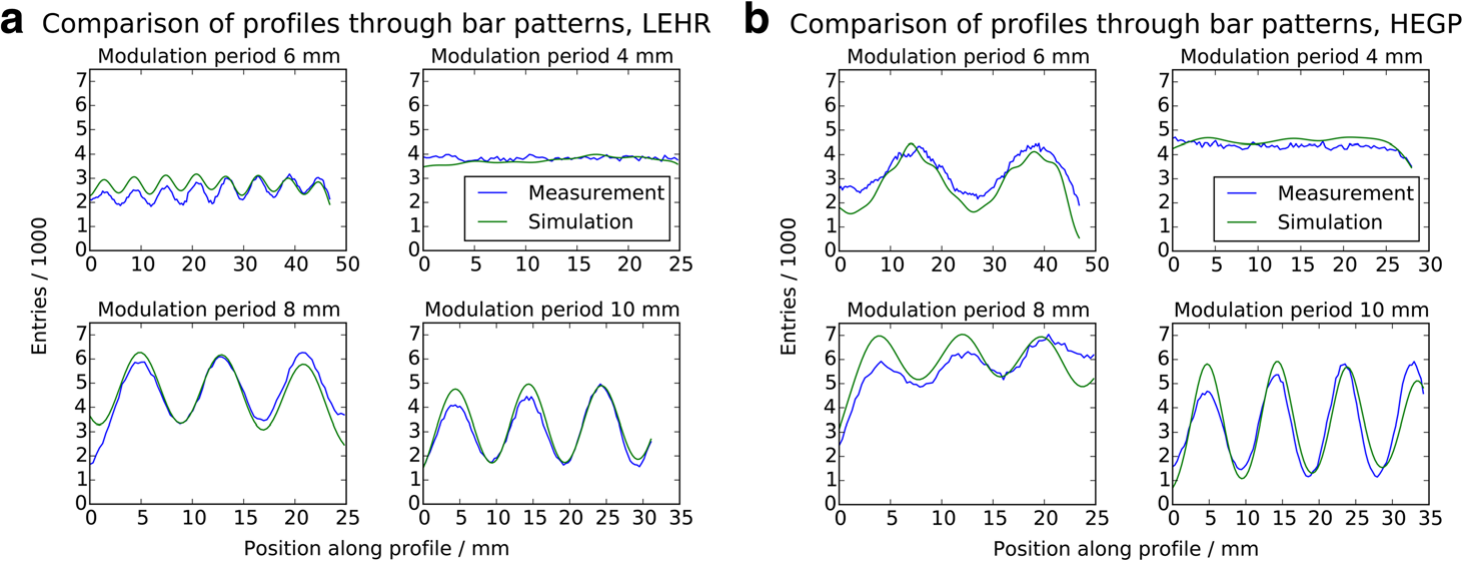
Profile plots of bar phantom. **a** Comparison of measured and simulated profiles through the modulation pattern of the bar phantom in the plane setup. **b** Low-energy, high-resolution collimator; bottom half: high-energy, general-purpose collimator. Simulated results include the spatial resolution model and were normalized to have the same maximum value as the measured profiles

A comparison of these profiles between measurement and simulation is presented in Fig. 7. The reproduction of the resolvable patterns is well modeled for either collimator, and one can more clearly identify the Moiré pattern exhibited by the 6-mm quadrant imaged with the HEGP collimator.

**Fig. 8:**
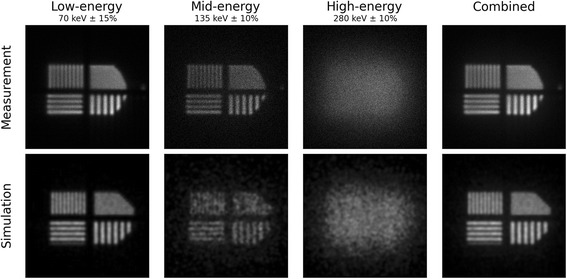
Bar phantom energy windows. Measured and simulated images in planar setup decomposed into contributions from three energy windows and their combination. Images show a sample with activity ratio *R*_*a*_ = 5.9 imaged through the low-energy, high-resolution collimator

Figure 8 shows the contributions to the final LEHR image from each of the energy windows. One can observe progressive deterioration of image quality with increasing energy both in measurement and in simulation.

**Fig. 9:**
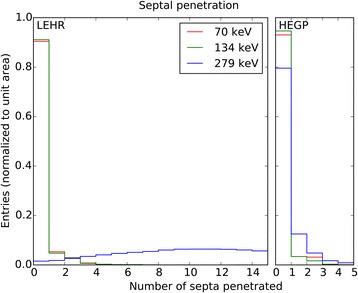
Septal penetration. Distributions of number of penetrated septa for both collimators and detected photons of three simulated emission energies

In the window at 135 keV, this is due to backscattered photons as demonstrated in the previous section, while image broadening in the 280-keV window could be a consequence of septal penetration. The relative contributions from these two windows to the combined image were 10 and 20%, respectively, while the window centered at 70 keV accounts for the bulk of the image. This composition depends on the time passed since production of ^197(m)^Hg, and earlier measurements would have had larger contributions from the higher energy windows. Using simulated data, the issue of septal penetration can be investigated further. Figure 9 shows the septal penetration rate for three source energies that was obtained from the simulation. While the penetration behavior of the 70- and 134-keV samples is very similar, the high-energy component has a tendency to penetrate more septa, as is to be expected. There is only a slight shift towards more penetration for the HEGP collimator, while the number of penetrated septa has its most probable value at 10 in the LEHR case.

### Tomographic imaging

**Fig. 10:**
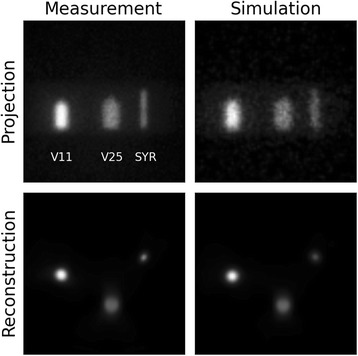
Tomographic images. Comparison of measured and simulated acquisitions in tomographic setup: top row: geometric means of the anterior and posterior projections; bottom row: SPECT reconstructions of central axial slice

Figure 10 shows the geometric mean of the anterior and posterior projections of the tomographic setup for measurement and simulations. The positions and relative intensities of the three sources agree quite well, but the simulated image shows a fair amount of statistical noise. While the neck structure of the vial sources is hinted at in the measured projection, it cannot be made out in the simulated one. The figure also shows the reconstructed central axial slice, and again, positions and intensities agree quite well overall. To investigate the spatial resolution of the reconstruction, profiles connecting the centroid source positions were extracted. The inter-source distances agree to within a few millimeters, while the inner diameters of the vial sources reconstructed from SPECT measurements are within 5%, and the simulated ones within 10%, of the physically measured values. In both cases, a slight underestimation occurs. The SYR source, on the other hand, is reconstructed with a width more than twice the actual value, indicating that the resolution limit is somewhere between the SYR and V11 diameters, i.e., between 5 and 19 mm.

## Discussion

The first application of radioactive mercury isotopes for nuclear medicine imaging was 60 years ago [1, 2]. However, radiomercury became unappealing due to neurotoxic side effects [5, 6]. These side effects were caused by the large amount of substance that was required for imaging due to the low specific activity of reactor produced radiomercury. Cyclotron-produced ^197(m)^Hg allows now imaging with high specific activity as wells as the availability of a large amount of the isomeric state ^197m^Hg that is very prospecting for therapeutic application [7]. ^197m^Hg has a half-life of 23.8 h and emits monoenergetic conversion electrons with energies of 150 keV (50%) and 119 keV (33%). These electrons have a maximal range in water of 0.3 mm [7, 16]. Hence, prospectively, a steep dose gradient is expected in tumors containing ^197m^Hg. Previous dose calculations revealed that the dose per decay from ^197(m)^Hg is 1.89 times greater than the dose from ^177^Lu in a spherical lesion with a mass of 10 g [17]. In addition, ^197m^Hg and ^197^Hg emit low-energy Auger electrons with a maximum range of about 1 μm that makes ^197(m)^Hg attractive to radiobiological cell experiments. Whereas future therapeutic applications are promising, in the first step, the imaging capabilities of ^197(m)^Hg were studied to evaluate if this isotope is valuable for molecular imaging or theranostic applications.

The scope of our work was to investigate the imaging capabilities of radiomercury ^197(m)^Hg under experimental conditions and to compare the results with Monte Carlo simulations. Due to the complex emission spectrum of ^197(m)^Hg with simultaneous emission of gammas and X-rays in the range of 50–300 keV, Monte Carlo simulations offer an option to explore the physical processes that determine imaging performance. Therefore, we used the software GATE that was previously used by other groups to simulate the imaging processes for SPECT and PET systems [11, 18–20].

Energy resolution of the Philips BrightView gamma camera was measured at four energies and found to not scale with the frequently assumed $1/\sqrt{E}$energy dependence. This behavior is caused by other sources of variation in output pulse amplitude beside statistical fluctuations, for example, nonuniform sensitivity of NaI crystal or nonuniform light collection efficiency [15]. A linear relationship was adopted instead, and the comparison of measured and simulated spectra (Fig. 4a) shows a good recreation of widths and positions of photo-peaks. There is also good agreement in the intermediate energy regions, mostly populated by background events and photons, which were inelastically scattered. The correct incorporation of this latter signal provides assurance that the material geometries have been modeled sufficiently accurately. The good representation of the mid-energy peak and the contribution of backscattered high-energy photons as shown in simulations (Fig. 5), in particular, demonstrate that the BSC was adequately described, despite a lack of detailed knowledge regarding its exact geometry. Concerning photo-peak amplitudes, one can see a good general agreement in Fig. 4a. While the lack of an absolute normalization makes it hard to ascertain which energy region is best represented in terms of intensity, the current scheme hints at a slight overrepresentation of the high-energy emissions, near-perfect agreement in the mid-energy domain, and reasonable correspondence for the low-energy cluster. Some discrepancies do, however, become apparent in the latter. While there is a marked shoulder structure at its high end (75–80 keV) in the measurement, this behavior is only hinted at in simulations. This region of the spectrum is populated by a Gamma emission from the ground state (77 keV), as well as *K*_*β*_ X-ray emissions from both isomers (average energies 80 and 78 keV). All of these were represented with 77 keV photons in the simulations, resulting in a lack of detail. Considering the energy window at 70 keV as a whole; however, the accuracy was judged sufficient to evaluate imaging capabilities. Lead X-rays from the collimator can interfere with gamma-ray emission in that energy window and were modeled in the simulation.

A realistically modeled spatial resolution is likely to be of greater importance for imaging studies. Extraction of the intrinsic resolution of the scintillator-PMT system via the method chosen here is limited by the range of energies, modulation directions, and positions considered. Detailed measurements of the pure intrinsic component with a uniformly illuminated multi-slit plate at different energies and orientations would have been preferable, but even in their absence, correspondence seems sufficient. Comparing modulation patterns in the plane setup (Fig. 7) revealed a very good agreement between measurement and simulation as well with the vendor specification. This comparison is made at minimal source-to-collimator distances, where the intrinsic component to system resolution is significant. At farther distances, collimator resolution becomes the limiting component. Resolution also compares reasonably well in this domain (Fig. 10), albeit including the obfuscating influence of scattered events in the tomographic setup. Downscatter corrections are important especially when using the low-energy window. Hence, for correct SPECT quantification of ^197(m)^Hg, attenuation and scatter correction models need to be implemented by camera manufacturers.

As far as ^197(m)^Hg imaging is concerned, one can see that it is largely dominated by emissions in the low-energy range around 70 keV. This is partly due to the higher emission yield in this region, which increases further as the sample ages. Another aspect favoring the low-energy range is its compatibility with LEHR collimators. Since they are usually designed for ^99m^Tc imaging, one would expect the mid-energy region near 134 keV to also be suitable for imaging, but this region contains a non-negligible contribution of backscattered high-energy photons. Low-energy emissions do suffer from greater attenuation, however, so it remains to be seen whether they can be usefully exploited in a realistic clinical scenario.

Our study has two limitations. On the one hand, no calibrated measurement device was available to direct measure the activity of ^197^Hg and ^197m^Hg. Hence, we could only estimate the activity by using the calibration factor of ^123^I for our dose calibrator. Nevertheless, we considered the activity ratio *R*_*a*_ that was measured precisely by gamma spectroscopy. On the other hand, no medium energy collimator was available for phantom measurements, although it could be expected that this collimator would be most suitable for imaging. Despite those methodical limitations, the main results were not affected.

^197m^Hg is attractive for therapeutic application due to the emission of low-energy Auger and conversion electrons. Imaging is possible with both radionuclides but especially with ^197^Hg. Hence, pure diagnostic tracers could be labeled with ^197^Hg alone to reduce the radiotoxicity for patients. This is an advantage in comparison to the beta-particle emitting radionuclide ^177^Lu that is used for therapy with only 17% gamma emission probability.

## Conclusions

In conclusion, we demonstrated well the feasibility of ^197(m)^Hg for molecular imaging. This was investigated by measurements and verified by Monte Carlo simulations. Thereby, the suitability of GATE for Monte Carlo simulation of a Philips BrightView camera was demonstrated. Molecular imaging of ^197(m)^Hg is a prerequisite for in vivo dosimetry during future application in the treatment of micrometastases and small tumors.

## Abbreviations

BSC: Backscatter compartment
HEGP: High-energy general-purpose
LEHR: Low-energy high-resolution
LSF: Line spread function
MTF: Modulation transfer function
PMMA: Polymethyl-methacrylate
